# Cytoprotection of Probiotic *Lactobacillus acidophilus* with Artificial Nanoshells of Nature-Derived Eggshell Membrane Hydrolysates and Coffee Melanoidins in Single-Cell Nanoencapsulation

**DOI:** 10.3390/polym15051104

**Published:** 2023-02-22

**Authors:** Sang Yeong Han, Duc Tai Nguyen, Beom Jin Kim, Nayoung Kim, Eunhye K. Kang, Ji Hun Park, Insung S. Choi

**Affiliations:** 1 Center for Cell-Encapsulation Research, Department of Chemistry, KAIST, Daejeon 34141, Republic of Korea; 2 Department of Chemistry, University of Ulsan, Ulsan 44776, Republic of Korea; 3 Department of Science Education, Ewha Womans University, Seoul 03760, Republic of Korea

**Keywords:** coffee melanoidins, eggshell membrane hydrolysates, *Lactobacillus acidophilus*, single-cell nanoencapsulation, probiotics

## Abstract

One-step fabrication method for thin films and shells is developed with nature-derived eggshell membrane hydrolysates (ESMHs) and coffee melanoidins (CMs) that have been discarded as food waste. The nature-derived polymeric materials, ESMHs and CMs, prove highly biocompatible with living cells, and the one-step method enables cytocompatible construction of cell-in-shell nanobiohybrid structures. Nanometric ESMH-CM shells are formed on individual probiotic *Lactobacillus acidophilus*, without any noticeable decrease in viability, and the ESMH-CM shells effectively protected *L. acidophilus* in the simulated gastric fluid (SGF). The cytoprotection power is further enhanced by Fe^3+^-mediated shell augmentation. For example, after 2 h of incubation in SGF, the viability of native *L. acidophilus* is 30%, whereas nanoencapsulated *L. acidophilus*, armed with the Fe^3+^-fortified ESMH-CM shells, show 79% in viability. The simple, time-efficient, and easy-to-process method developed in this work would contribute to many technological developments, including microbial biotherapeutics, as well as waste upcycling.

## 1. Introduction

One-step film construction, referring to the process that involves mixing of complimentary coating components and generation of interface-active species prior to film formation, significantly simplifies film fabrication procedures, particularly compared with sequential deposition approaches (e.g., layer-by-layer, LbL) [1,2,3,4], and eliminates the repeated deposition steps if it affords continuous film growth. Its notable examples include material-independent coating of tannic acid (TA) and Fe^3+^, where use of the preformed TA-Fe^3+^ sol enables much simplified procedures with (semi)-continuous film growth [5,6,7,8,9]. In-situ generation of interface-active TA-Fe^3+^ species, for example, the one inspired by iron gall ink [10,11,12], would be another strategy for the one-step, continuous film formation [10,11,12,13,14].

**Figure 1:**
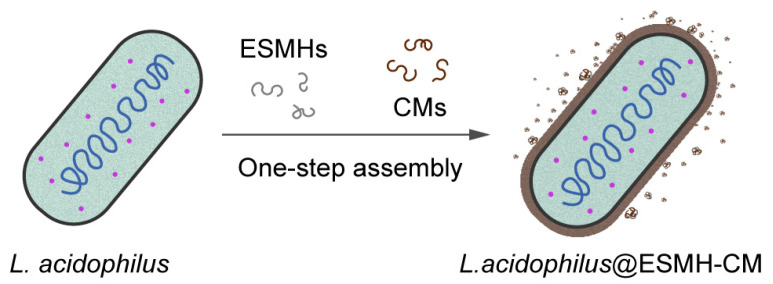
Schematic for formation of ESMH-CM shells on individual *Lactobacillus acidophilus*.

The one-step approach is especially beneficial in the field of single-cell nanoencapsulation (SCNE) [15,16,17,18,19,20], where cytoprotective nanometric-shells are constructed on individual living cells, leading to the creation of cell-in-shell nanobiohybrids. Its procedural simplicity also would be seamlessly coupled with the existing industrial processes, for instance, for fabrication of probiotic capsules, as well as enhancing biocompatibility with living cells, probiotics in this study. As a related work, tea polyphenols and poly(*N*-vinylpyrrolidone) (PVPON) were added simultaneously to an aqueous cell suspension for cytocompatible formation of cell-in-shell structures with a polymeric complex of tea polyphenols and PVPON [21], as an alternative to the LbL-based SCNE with TA and PVPON [22]. It is envisaged that the identification of other nature-derived materials, structurally different from polyphenolics, would further upscale the usability and applicability of one-step film construction in the practical applications as well as in the research domain of single-cell surface engineering including SCNE [23]. In this work, we constructed a cell-in-shell structure of probiotic *Lactobacillus acidophilus* by one-step, cytocompatible formation of the cytoprotective shells comprising nature-derived eggshell membrane hydrolysates (ESMHs) and coffee melanoidins (CMs) (Figure 1).

ESMHs, hydrolyzed ESMs, have recently been suggested as nature-derived biomaterials in the LbL-based formation of thin films [24]. In consideration of high content of proteins (80–85%) in ESMs, including collagens (types I, V, and X) and glycosaminoglycans, ESMHs could serve as extracellular matrix-mimetic materials in the manipulation of living cells. The LbL-SCNE methods with ESMH-TA [25] and ESMH-CM pairs [26] have previously been demonstrated to show the great cytocompatibility of ESMHs. Utilization of ESMHs as biomaterials also would contribute to the waste upcycling in the aspect of circular economy; eggshells, comprising about 11% of egg weight, have been discarded as food waste [27,28]. In the aspect of waste upcycling, spent coffee grounds also have been explored as a sustainable solution in various fields, such as photothermal materials in sterilization, fertilizers and soil conditioners in the agricultural industry, raw materials for biodiesel and biochar production, and sorbents in water management [29,30,31,32]. CMs, a product of the Maillard reaction during coffee-roasting process, exhibit antioxidant activity as well as containing biologically active molecules [33]. Aside from potential use as nutraceuticals [34], CMs, extracted from spent coffee ground, form thin films with Fe^3+^, which has been utilized in SCNE [35]. The combined use of ESMHs and CMs as next-generation biomaterials would widen the material scope in biomedical engineering and related fields, assisted by the development of simple, one-step methods for forming ultrathin films and shells.

## 2. Materials and Methods

### 2.1. One-Step Formation of ESMH-CM Films and Shells on Abiotic Substrates

The ESMHs and CMs were prepared according to our previous reports [26,35]. The stock solution of ESMHs or CMs was made to the final concentration of 2 mg/mL in a sodium chloride (NaCl) solution (50 mM). Prior to use, gold substrates were cleaned with ethanol and acetone. The cleaned gold substrates were immersed in a 1:1 mixture of the ESMH and CM stock solutions (500 μL each), stirred at 120 rpm for 3 h, washed with deionized (DI) water, and dried under a stream of argon gas. The same protocol was employed for other flat substrates (silver, aluminum, copper, nickel, tin, titanium, silicon, stainless steel, poly(acrylic acid), polycarbonate, polyethylene, polyurethane, and polytetrafluoroethylene). Calcium carbonate (CaCO_3_) particles were prepared by mixing 4 mL of an aqueous poly(sodium 4-styrenesulfonate) solution (PSS, MW: ca. 70,000, 2 mg/mL), 48 µL of an aqueous sodium carbonate solution (Na_2_CO_3_, 1 M), and 96 µL of an aqueous calcium chloride solution (CaCl_2_, 1 M) under vigorous stirring for 40 s, incubating for 7 min at room temperature, and calcinating at 450 °C for 2 h. ESMH-CM shells were formed on the resulting CaCO_3_ particles with a 1:1 mixture of the ESMH and CM stock solutions (500 µL each).

### 2.2. Single-Cell Nanoencapsulation (SCNE) and Characterizations

A single colony of *Saccharomyces cerevisiae*, picked from the YPD agar plate, was cultured for 30 h in a YPD broth medium at 33 °C. After washing with DI water, *S. cerevisiae* were immersed for 3 h in a 1:1 mixture of the ESMH and CM stock solutions (500 μL each) and washed with DI water three times. The same SCNE protocol was employed for *L. acidophilus* and *Levilactobacillus brevis*, after culturing for 24 h in an MRS broth medium at 33 °C. For viability assay of *S. cerevisiae*, 5 μL of the stock solution of fluorescein diacetate (FDA, 10 mg/mL in acetone) and 2 μL of an aqueous solution of propidium iodide (PI, 1 mg/mL) were added to a *S. cerevisiae* suspension (1 mL), and the mixture was incubated for 15 min at 33 °C. SYTO 9 was used instead of FDA for the viability assay of *L. acidophilus* and *L. brevis.* Both 2 μL of the SYTO 9 stock solution (3.34 mM in DMSO) and 2 μL of the PI stock solution (20 mM in DMSO) were added to a cell suspension (1 mL). The mixture was then incubated for 20 min at 33 °C. To form the ESMH-CM[Fe^3+^] shell, ESMH-CM-encapsulated cells (denoted as cell@ESMH-CM) were immersed in an aqueous solution of FeCl_3_ (10 mM) for 30 min. The $t_{-2.0}^{{\mathrm{OD}}_{600}}$ values were calculated based on the results of cell culture in the MRS broth medium. In short, 1 mL of an aqueous cell suspension (*L. acidophilus*, *L. acidophilus*@ESMH-CM, or *L. acidophilus*@ESMH-CM[Fe^3+^], ${\mathrm{OD}}_{600}$ = 0.15) was added to 150 mL of the MRS broth medium (final ${\mathrm{OD}}_{600}$ = 0.001) and incubated at 33 °C. The 100 μL of the culture mixture was picked at the predetermined time, and the cell density was measured at 600 nm with a microplate reader. Linear fitting of $\ln\left({\mathrm{OD}}_{600}\right)$, from −4.0 to +1.0, with incubation time (in hour) gave $t_{-2.0}^{{\mathrm{OD}}_{600}}$, the time for $\ln\left({\mathrm{OD}}_{600}\right)$ of −2.0. For cytoprotection studies, cells were suspended in 1 mL of an aqueous polyethylenimine solution (PEI, 0.5, 1, 10, or 50 mg/mL) for 30 min or 1 mL of an aqueous TA solution (1, 5, 10, 25, or 50 mg/mL) for 1 h. The simulated gastric fluid (SGF) was prepared by dissolving NaCl (0.2% (*w*/*v*)) and pepsin (3 mg/mL) in DI water, followed by pH adjustment to 2 with 1 M HCl. The cells were suspended in the SGF solution and incubated for 2 h at 37 °C for cytoprotection studies against the SGF.

## 3. Results and Discussion

### 3.1. One-Step Formation of ESMH-CM Films and Shells on Abiotic Substrates

**Figure 2:**
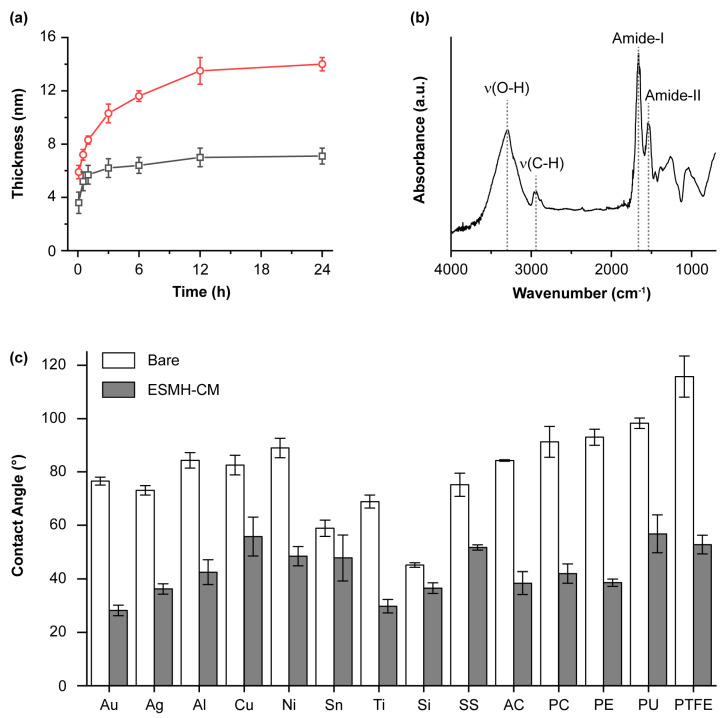
(**a**) Thickness of ESMH-CM films: (red) 50-mM NaCl and (gray) no NaCl. (**b**) FT-IR spectrum of the ESMH-CM film on a gold substrate. (**c**) Static water contact angles (white) before and (gray) after ESMH-CM-film formation. Au: gold; Ag: silver; Al: aluminum; Cu: copper; Ni: nickel; Sn: tin; Ti: titanium; Si: silicon; SS: stainless steel; AC: poly(acrylic acid); PC: polycarbonate; PE: polyethylene; PU: polyurethane; PTFE: polytetrafluoroethylene.

Prior to SCNE, we investigated the feasibility of one-step film formation with ESMH-CM complex and optimized the reaction conditions, with a gold substrate as a model. The concentrations of ESMHs and CMs were set to be 1 mg/mL each. The ellipsometric thickness measurement, after 3 h of reaction, indicated that a film was formed with 6.2 nm of thickness. It was also found that the addition of NaCl to the coating mixture increased film thickness, and 50 mM of NaCl was chosen as an optimized concentration in this study. The film thickness significantly increased to 10.3 nm with 50 mM of NaCl as an additive after 3 h of reaction (166% increase). Detailed thickness analysis showed that the film growth stopped after about 3 h without NaCl (thickness: 7.1 nm), but in stark contrast, the ESMH-CM film grew continuously at least up to 24 h (thickness: 14.0 nm) (Figure 2a). As a control, we used only a single component (ESMHs or CMs) in the film formation. The thickness analysis showed that 1.6-nm-thick and 0.8-nm-thick films were formed with ESMHs and CMs, respectively, under the same conditions, confirming the significance of pre-association of ESMHs and CMs in solution (Figure S1). On the other hand, it was observed that the pre-mixed ESMH-CM pair also could be utilized in the LbL-type film formation: film thickness increased in a linear fashion, with 2.8 nm per 10 min of incubation, making 28 nm thick films after 10 deposition cycles (Figure S2).

The films formed after 3 h of reaction were characterized by Fourier transform infrared (FT-IR) spectroscopy, X-ray photoelectron spectroscopy (XPS), field-emission scanning electron microscopy (FE-SEM), and atomic force microscopy (AFM). The signature bands for ESMHs and CMs at 1666 (amide-I stretching) and 1550 cm^−1^ (amide-II stretching), in addition to the *v*(C−H) band at 2960 cm^−1^ and *v*(O−H) band at 3297 cm^−1^, in the FT-IR spectrum indicated the successful formation of ESMH-CM films (Figure 2b), further supported by the XPS analysis showing C 1s and N 1s peaks (Figure S3a). The C 1s XPS peak was deconvoluted into three peaks at binding energies of 283.6 (C−C and C−H), 284.8 (C−O and C−N), and 287.0 eV (C=O and C=N), and the one for the N 1s peak was further deconvoluted into two peaks at 398.4 (C−NH) and 399.1 eV (O=C−N), additionally providing evidence for the presence of carbohydrates and peptides in the film (Figure S3b,c) [35,36]. The FE-SEM and AFM analysis showed that the ESMH-CM films were composed of nanoparticulates, clearly distinct from the bare gold surface (Figure S3d,e). The water-contact angle measurements also indicated the successful formation of ESMH-CM films: the contact angle was changed to 28.2° from 76.6°, after film formation.

We also examined whether our one-step method for ESMH-CM-film formation was universal, applicable to interface engineering of various different substrates, including silver, aluminum, copper, nickel, tin, titanium, silicon, stainless steel, poly(acrylic acid), polycarbonate, polyethylene, polyurethane, and polytetrafluoroethylene (PTFE), in addition to gold. The water-contact angle measurements clearly showed that the one-step method was material-independent (Figure 2c). The water-contact angles of all the substrates tested were changed to be below 60° after film formation, regardless of their intact angles: for example, the contact angle of PTFE was changed to 52.8° from 115.7°. In addition to the flat substrates, our one-step method was employed for the construction of core-shell structures in particle engineering, exemplified with CaCO_3_ and amine-terminated silica (SiO_2_) particles. Changes in the zeta (ζ) potential indicated the formation of ESMH-CM shells on the particles: −15.7 eV from +4.1 eV for CaCO_3_ particles (diameter: 2–4 μm) and −35.5 eV from +44.3 eV for SiO_2_ particles (diameter: 3.92 μm) (Figure S4a). It is of note that the shell formation occurred regardless of the surface charge of particles. The formation of ESMH-CM shells was visualized with a rhodamine-linked ESMH (ESMH_TAMRA, λ_emission_: 575 nm) [25,26] by confocal laser-scanning microscopy (CLSM) (Figure S4b). The ability to form shells on individual particles under biocompatible synthetic conditions suggested the potential of our system in the SCNE of living cells.

### 3.2. One-Step SCNE of S. cerevisiae

After confirming the one-step formation of films and cells on abiotic substrates with ESMHs and CMs, the protocol was applied to the SCNE with *S. cerevisiae* as a model. *S. cerevisiae* was chosen for investigation of cytocompatibility of our method and cytoprotectability of the formed ESMH-CM shell, because of the availability of numerous reference reports on the SCNE of *S. cerevisiae* [37,38].

**Figure 3:**
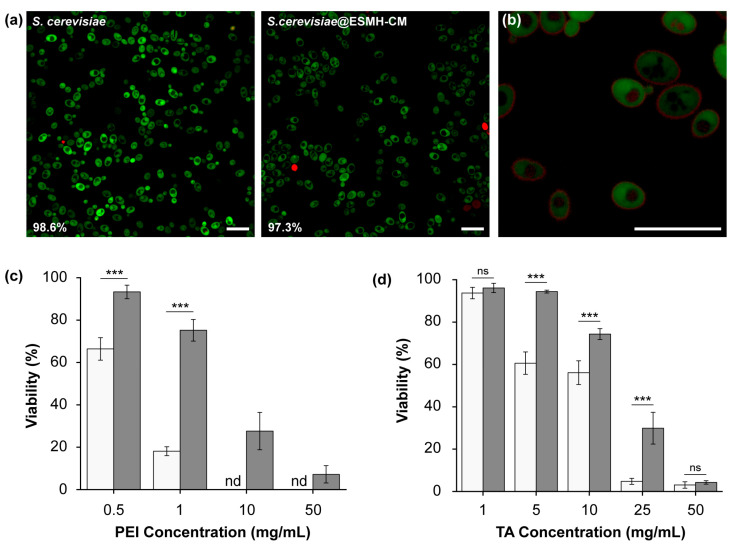
(**a**) Viability: CLSM images of *S. cerevisiae* before and after formation of ESMH-CM shells. Green: live; red: dead. Scale bar: 20 μm. (**b**) CLSM image of FDA-treated *S.cerevisiae*@ESMH_TAMRA-CM. Scale bar: 20 μm. (**c**,**d**) Cytoprotection against (**c**) PEI and (**d**) TA: (white) bare *S. cerevisiae* and (gray) *S.cerevisiae*@ESMH-CM. Data are expressed as mean ± standard deviation. Statistical significance was analyzed by Student’s *t*-test. *** *p* < 0.001; nd: not detected; ns: not significant.

*S. cerevisiae* was incubated for 3 h in a 50 mM NaCl solution of ESMHs (1 mg/mL) and CMs (1 mg/mL), leading to the construction of *S.cerevisiae*@ESMH-CM. The cell viability, after SCNE, was analyzed with FDA (λ_emission_: 521 nm; for live cells) and PI (λ_emission_: 636 nm; for dead cells). FDA is a membrane-permeable, fluorogenic viability-probe that measures both enzymatic activity and membrane integrity, and PI is a membrane-impermeable, nucleic-acid-intercalating agent that is commonly used to detect dead cells. The CLSM images showed that most *S.cerevisiae*@ESMH-CM cells were viable (Figure 3a), and the quantitative analysis showed 98.7% of %viability (calculated by dividing the viability of *S.cerevisiae*@ESMH-CM (97.3 ± 0.9%) by the viability of intact, bare *S. cerevisiae* as a reference (98.6 ± 1.0%)), indicating no noticeable harm to the cells. That is, the viability assay confirmed that the one-step shell formation with ESMHs and CMs was extremely cytocompatible. The ESMH-CM shells on *S. cerevisiae* were visualized with use of ESMH_TAMRA by CLSM, which showed green/red core/shell structures of FDA-stained *S. cerevisiae* (Figure 3b).

Cytoprotectability of the ESMH-CM shells on *S. cerevisiae* was tested and demonstrated with PEI (branched, MW: 25,000). The viability of bare *S. cerevisiae* decreased significantly with the PEI concentration, after 30 min of incubation in a PEI solution (pH 7, in DI water) (Figure 3c): for instance, the viability was calculated to be 18.1 ± 2.1% in the case of 1 mg/mL, and no bare *S. cerevisiae* survived the PEI concentration of 10 mg/mL. In striking contrast, the viability of *S.cerevisiae*@ESMH-CM was 75.2 ± 5.1% (ca. 5-fold increase in viability) for 1 mg/mL of PEI, and 27.6 ± 8.8% of *S.cerevisiae*@ESMH-CM was viable in the case of 10 mg/mL of PEI. In addition, the enhanced viability against various concentrations of TA was also observed for *S.cerevisiae*@ESMH-CM (Figure 3d). The SCNE results with *S. cerevisiae* arguably confirmed that our one-step ESMH-CM method created cytoprotective shells in the cytocompatible manner, which is the primary requirement of the first-generation cell-in-shell nanobiohybrids or artificial spores [19,20]. It was also noticeable that the ESMH-CM shell of only ca. 10 nm in thickness had such cytoprotective power. The cytoprotectability and durability of the shells could be enhanced further by forming ESMH-CM shells for a longer time than 3 h and/or repeating the shell-forming process. For instance, the thickness of ESMH-CM films on gold increased to 67.5 nm after 10 cycles of the film deposition ([ESMH] = [CM] = 1 mg/mL; 3 h of reaction) (Figure S5).

### 3.3. One-Step SCNE of Probiotic L. acidophilus and L. brevis

The ESMH-CM shells were formed on *L. acidophilus*, producing *L. acidophilus*@ESMH-CM. *L. acidophilus* is a gram-positive, microaerophilic probiotic bacterium, naturally present in the gastrointestinal (GI) tract, vagina, and others. It is one of the major probiotic species in commercialized products, such as yogurt and probiotic capsules, along with *L. bulgaricus*, *Streptococcus thermophilus*, and *Bifidobacterium bifidum*.

Innumerable encapsulation methods for *L. acidophilus* (and also other probiotics) have been attempted and reported to enhance the survival during food/nutraceutical processing as well as against harsh conditions in the stomach and GI tract [39,40,41]. In addition to the long-pursued microencapsulation approach [42,43], recent research efforts have intensively been devoted to the formation of nanometric shells on probiotics in SCNE [44,45,46]. Notable examples include the recent utilization of TA-Fe^3+^ nanoshells [47,48,49] for cytoprotective SCNE of anaerobic *Bacteroides thetaiotaomicron* [50] and *L. casei* [51] for potential development of microbial biotherapeutics, in addition to the autonomous nanoencapsulations of *L. rhamnosus* with polydopamine [52] and engineered *S. thermophilus* with hyaluronic acid [53]. Nanoshells of metal–organic frameworks and silica nanoparticles also have been used for potential cytoprotection of *L. acidophilus*, *B. infantis*, and *B. breve* [54,55]. Other endeavors in this direction involve the LbL construction of polyelectrolyte multilayers (PEMs), exemplified by the PEM shells on *L. acidophilus* [56], *L. rhamnosus* [57], *L. pentosus* [58], *L. plantarum* [59], and *Bacillus coagulans* [60].

**Figure 4:**
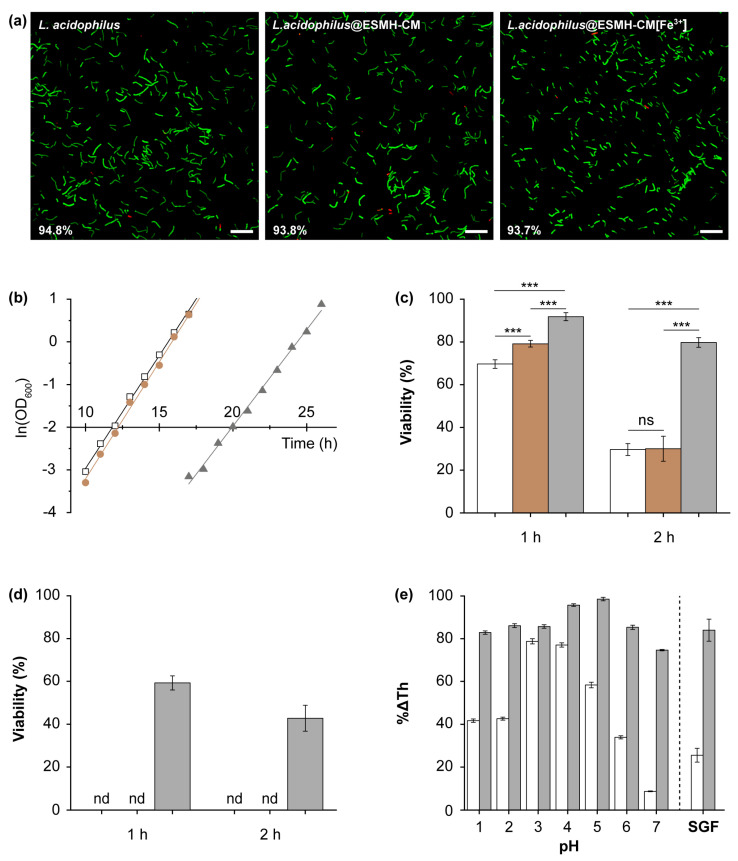
(**a**) Viability of *L. acidophilus*: CLSM images of (left) bare *L. acidophilus*, (middle) *L. acidophilus*@ESMH-CM, and (right) *L. acidophilus*@ESMH-CM[Fe^3+^]. Scale bar: 20 μm. (**b**) Linear-fitted plots from −4.0 to +1.0 of ln(OD_600_) of (open black square) *L. acidophilus*, (brown circle) *L. acidophilus*@ESMH-CM, and (gray triangle) *L. acidophilus*@ESMH-CM[Fe^3+^] (**c**) Cytoprotection of *L. acidophilus* against SGF: (white) bare *L. acidophilus*, (brown) *L. acidophilus*@ESMH-CM, and (gray) *L. acidophilus*@ESMH-CM[Fe^3+^]. Data are expressed as mean ± standard deviation. Statistical significance was analyzed by Student’s *t*-test. *** *p* < 0.001; ns: not significant. (**d**) Cytoprotection of *L. brevis* against SGF: (white) bare *L. brevis*, (brown) *L. brevis*@ESMH-CM, and (gray) *L. brevis*@ESMH-CM[Fe^3+^]. nd: not detected. (**e**) Film degradation after 2 h of incubation at various pHs and in SGF: (white) ESMH-CM and (gray) ESMH-CM[Fe^3+^] films. %ΔTh: percent film thickness with initial film thickness as a reference. Data are expressed as mean ± standard deviation.

*L. acidophilus*@ESMH-CM was constructed by simply incubating *L. acidophilus* in a 50-mM NaCl solution of ESMHs (1 mg/mL) and CMs (1 mg/mL) for 3 h. The viability of *L. acidophilus*@ESMH-CM, after SCNE, was investigated with SYTO 9 (λ_emission_: 503 nm) and PI. SYTO 9 is a nucleic-acid stain for bacteria [61,62], and the combination of SYTO 9 and PI has widely been used for bacterial-viability assays [63]. The assay showed 98.9% of %viability for *L. acidophilus*@ESMH-CM (viability: showing 93.8 ± 1.4%) with pristine *L. acidophilus* as a reference (viability: 94.8 ± 2.3%) (Figure 4a). In addition to the construction of *L. acidophilus*@ESMH-CM, inspired by our previous report on Fe^3+^-mediated shell augmentation [24,28], we formed Fe^3+^-fortified *L. acidophilus*@ESMH-CM, denoted as *L. acidophilus*@ESMH-CM[Fe^3+^], by incubating *L. acidophilus*@ESMH-CM for 30 min in an aqueous solution of FeCl_3_ (10 mM). No decrease in viability was observed after Fe^3+^ fortification (viability: 93.7 ± 1.3%). It was also verified that the Fe^3+^-mediated shell augmentation prolonged the lag phase of *L. acidophilus*, implying that the Fe^3+^ fortification would be another chemical tool for manipulation and control of cellular activities and metabolism (Figure 4b). Quantitatively, the $t_{-2.0}^{{\mathrm{OD}}_{600}}$ values [64] were calculated to be 11.8, 12.2, and 19.9 h for pristine *L. acidophilus*, *L. acidophilus*@ESMH-CM, and *L. acidophilus*@ESMH-CM[Fe^3+^], respectively.

Considering the importance of sustained survival after passage through the stomach in the development and formulation of probiotic capsules, viabilities of bare *L. acidophilus*, *L. acidophilus*@ESMH-CM, and *L. acidophilus*@ESMH-CM[Fe^3+^] were measured and compared after incubation in SGF (pH 2) (Figure 4c). After 1 h of incubation, the viability of bare *L. acidophilus* was calculated to be 69.5 ± 1.7%, in comparison with 78.3 ± 1.5% for *L. acidophilus*@ESMH-CM and 92.1 ± 1.3% for *L. acidophilus*@ESMH-CM[Fe^3+^], signifying the Fe^3+^-fortified *L. acidophilus*@ESMH-CM would be protected effectively during the passage though the stomach. The cytoprotection of *L. acidophilus*@ESMH-CM[Fe^3+^] was much more discernable for 2 h of incubation. The viability decreased significantly to 29.6 ± 2.8% and 30.0 ± 5.8% for bare *L. acidophilus* and *L. acidophilus*@ESMH-CM, respectively (not significant between the two values, based on Student’s *t*-test). In stark contrast, 78.5 ± 1.3% of %viability was observed for *L. acidophilus*@ESMH-CM[Fe^3+^]. The results clearly confirmed the cytoprotection capability of ESMH-CM and ESMH-CM[Fe^3+^] shells, suggesting great potential in the construction of probiotic capsules and/or microbial biotherapeutics [65]. It is yet to mention that the cytoprotection degree of ESMH-CM-based shells was species-dependent in the absolute sense. For example, *L. brevis*, a species in vaginal microbiota [66], was observed to be more labile than *L. acidophilus* in SGF. Neither bare *L. brevis* nor *L. brevis*@ESMH-CM survived after 1 h of incubation, whereas 42.7 ± 6.0% of *L. brevis*@ESMH-CM[Fe^3+^] were viable even after 2 h of incubation in SGF (Figure 4d). Although the value (ca. 43 %) was less than the %viability for *L. acidophilus* (ca. 79%), the ESMH-CM[Fe^3+^] showed reasonable protection of *L. brevis* against the attack of SGF.

It could be thought that the observed enhancement in cell viability against SGF was attributed to the stability of ESMH-CM-based shells under acidic conditions, and a model study was carried out. The ESMH-CM and ESMH-CM[Fe^3+^] films on gold were incubated for 2 h at various pH values (1, 2, 3, 4, 5, 6, and 7) as well as in SGF, and % decrease in film thickness (%ΔTh) was calculated after ellipsometric-thickness measurements (Figure 4e). The ESMH-CM film was observed to be stable at pH 3 and 4, but not at pH 2 and below. Accordingly, about 24% of the ESMH-CM film remained after 2 h of SGF treatment. In contrast, the Fe^3+^ fortification made the film much more durable at all the pHs tested, as well as in SGF. For example, 84% of the ESMH-CM[Fe^3+^] film was maintained after 2 h of SGF treatment, in a good agreement with the viability studies above. Of interest, the ESMH-CM[Fe^3+^] film decreased to about 74% in thickness at pH 7, implying that the nanoencapsulated probiotics could inhabit the gut epithelium after protected passage of the stomach.

## 4. Conclusions

In summary, we developed a simple but scalable method for constructing ultrathin films and shells, based on nature-derived biomaterials—eggshell membrane hydrolysates (ESMHs) and coffee melanoidins (CMs). The process developed proved extremely biocompatible with living microbial cells, applied seamlessly to probiotic bacteria, *Lactobacillus acidophilus* (in gut microbiota) and *Levilactobacillus brevis* (in vaginal microbiota). The ESMH-CM shells protected the probiotic bacteria in the simulated gastric fluid, suggesting potential in the probiotics nanoencapsulation. The cytoprotectability was further augmented by the Fe^3+^-mediated cross-linking of ESMH-CM shells. Considering that multivalent metal cations are present in body fluids, such as Fe^3+^, Ca^2+^, Mg^2+^, Zn^2+^, and Cu^2+^, the shell augmentation also could occur autonomously in the body, which is our next research thrust. In addition, our formulation might be combined with mineral supplements for shell augmentation during oral administration. Furthermore, in the aspect of biomaterials, ESMHs and CMs would add to the set of nature-derived biocompatible materials for fabrication of nanobiohybrid structures, with a characteristic of waste upcycling.

## Data Availability

Not applicable.

## Supplementary Materials

The following supporting information can be downloaded at: https://www.mdpi.com/article/10.3390/polym15051104/s1, Figure S1: Graph of film thickness after 3 h of incubation (ESMHs, CMs, and ESMH-CM). Figure S2: Graph of film thickness vs. number of depositions. Figure S3: XPS spectrum, and FE-SEM and AFM images of ESMH-CM films. Figure S4: Characterizations of particle@ESMH-CM. Figure S5: Graph of film thickness vs. the number of depositions (3 h of incubation).

## Abbreviations

AC	poly(acrylic acid)
AFM	atomic force microscopy
CaCl_2_	calcium chloride
CaCO_3_	calcium carbonate
CLSM	confocal laser-scanning microscopy
CM	coffee melanoidin
DMSO	dimethylsulfoxide
ESMH	eggshell membrane hydrolysate
FDA	fluorescein diacetate
FE-SEM	field-emission scanning electron microscopy
FT-IR	Fourier-transform infrared
GI	gastrointestinal
LbL	layer-by-layer
NaCl	sodium chloride
Na_2_CO_3_	sodium carbonate
PC	polycarbonate
PE	polyethylene
PEI	polyethylenimine
PI	propidium iodide
PSS	poly(sodium 4-styrenesulfonate)
PTFE	polytetrafluoroethylene
PU	polyurethane
PVPON	poly(*N*-vinylpyrrolidone)
SCNE	single-cell nanoencapsulation
SGF	simulated gastric fluid
SS	stainless steel
SiO_2_	Silica
TA	tannic acid
TAMRA	carboxytetramethylrhodamine
YPD	yeast-extract-peptone-dextrose
XPS	X-ray photoelectron spectroscopy
