# Supplementary material for: Cytoprotection of Probiotic Lactobacillus acidophilus with Artificial Nanoshells of Nature-Derived Eggshell Membrane Hydrolysates and Coffee Melanoidins in Single-Cell Nanoencapsulation
Source: Polymers (Basel). 2023 Feb 22;15(5):1104. doi: 10.3390/polym15051104 (PMC10007236; doi:10.3390/polym15051104)
Supplement: Supplementary file 1 [file polymers-15-01104-s001.zip › polymers-2205053-supplementary.pdf]

Supplementary Materials: Cytoprotection of Probiotic *Lactobacillus acidophilus* with Artificial Nanoshells of Nature-Derived Eggshell Membrane Hydrolysates and Coffee Melanoidins in Single-Cell Nanaencapsulation

Sang Yeong Han[1], Duc Tai Nguyen[1], Beom Jin Kim[2,*], Nayoung Kim[1], Eunhye K. Kang[1], Ji Hun Park[3], and Insung S. Choi[1,*]

[1] Center for Cell-Encapsulation Research, Department of Chemistry, KAIST, Daejeon 34141, Korea
[2] Department of Chemistry, University of Ulsan, Ulsan 44776, Korea
[3] Department of Science Education, Ewha Womans University, Seoul 03760, Korea

CONTENTS

- Experimental Section.
- **Figure S1**. Graph of film thickness after 3 h of incubation (ESMHs, CMs, and ESMH-CM).
- **Figure S2.** Graph of film thickness vs. number of depositions.
- **Figure S3.** XPS spectrum, and FE-SEM and AFM images of ESMH-CM films.
- **Figure S4.** Characterizations of particle@ESMH-CM.
- **Figure S5.** Graph of film thickness vs. the number of depositions (3 h of incubation).

Experimental Section

Materials. Sodium chloride (NaCl, 99.5% Daejung Chemicals), sodium hydroxide (NaOH, 95%, Junsei), calcium chloride dihydrate ($CaCl_2{\cdot}2H_2O$, ≥99.0%, Sigma-Aldrich), sodium carbonate (Na_2CO_3, ≥99.5%, Sigma-Aldrich), iron(III) chloride hexahydrate ($FeCl_3{\cdot}6H_2O$, ≥98.0%, Sigma-Aldrich), poly(sodium 4-styrenesulfonate) (PSS, Mw ~70,000, Sigma-Aldrich), polyethylenimine, branched (PEI, average Mw ~25,000, Sigma-Aldrich), tannic acid (TA, Sigma-Aldrich), hydrogen chloride solution (HCl, 35%, Junsei), ethanol (95.0%, Samchun Chemicals), acetone (99.5%, Samchun Chemicals), dimethyl sulfoxide (DMSO, ≥99.0%, Sigma-Aldrich), fluorescein diacetate (FDA, Sigma-Aldrich), propidium iodide (PI, Sigma-Aldrich), SYTO^{TM} 9 green fluorescent nucleic acid stain (Thermo Fisher Scientific), pepsin from porcine gastric mucosa (≥400 units/mg, Sigma-Aldrich), *S. cerevisiae* (ATCC 18824 from the Korean Collection for Type Cultures (KCTC)), *L. acidophilus* (ATCC 4356 from the American Type Culture Collection (ATCC)), *L. brevis* (ATCC 14869 from the ATCC), yeast-extract-peptone-dextrose (YPD) broth (Duchefa Biochemistry), YPD agar (Duchefa Biochemistry), *Lactobacilli* MRS broth (BD), *Lactobacilli* MRS agar (BD), silica particles, amino-functionalized (diameter: 3.92 µm, Microparticles GmbH), buffer solutions (pH 1, 2, 3, 4, 5, 6, and 7, Samchun Chemicals), silver foil (Ag, 99.9%), aluminum foil (Al, 99.997%, Alfa Aesar), copper foil (Cu, 99.95%, Alfa Aesar), nickel (Ni, 99.5%, Alfa Aesar), tin foil (Sn, 99.8%, Alfa Aesar), stainless steel (SS, Fe:Cu:Ni; 70:19:11 wt%, Alfa Aesar), and titanium foil (Ti, 99.5%, Alfa Aesar) were used as received. Polycarbonate (PC), polyethylene (PE), polypropylene (PP), polyurethane (PU), and polytetrafluoroethylene (PTFE) substrates were purchased at an internet market (ENGP, Korea). Deionized (DI) water (18.3 MΩ cm) from Milli-Q Direct 8 (Millipore) was used.

Characterizations. The ellipsometric thickness was measured with an Elli-SE spectroscopic ellipsometer (Ellipso Technology). At least five independent points of each sample were measured, and average value (with at least 20 measurements) were recorded. Field-emission scanning electron microscopy (FE-SEM) images were obtained with an Inspect F50 microscope (FEI) with an accelerating voltage of 5 kV or 10 kV, after sputter-coating with platinum. Atomic force microscopy (AFM) image was obtained with a NanoWizard 4 XP Bioscience AFM (JPK), in the QI mode using an HQ:NSC15/Al BS tip (MikroMasch). X-ray photoelectron spectroscopy (XPS) spectra were acquired with a Sigma Probe (Thermo VG Scientific). Fourier-transform infrared (FT-IR) spectra were recorded with a nitrogen-purged Thermo Nicolet Nexus FT-IR spectrophotometer. Static water contact angle measurements were performed using a Phoenix 300 goniometer (Surface Electro Optics Co.) equipped with a video camera. The static contact angle of a 3-µL water droplet was measured at three different locations on each sample, and average value were recorded. The confocal laser-scanning microscopy (CLSM) images were carried out with an LSM 700 (Carl Zeiss). The ζ-potential was measured with a Zetasizer Nano ZS (Malvern). The cell density was measured with a microplate reader (SpectraMax iD5, Molecular Devices).

One-Step Formation of ESMH-CM Films and Shells. The stock solution of ESMHs or CMs was made to the final concentration of 2 mg/mL in a NaCl solution (50 mM). Prior to use, gold

substrates were cleaned with ethanol and acetone. The cleaned gold substrates were immersed in a 1:1 mixture of the ESMH and CM stock solutions (500 μL each), stirred at 120 rpm for 3 h, washed with DI water, and dried under a stream of argon gas. The same protocol was employed for other flat substrates. $CaCO_3$ particles were prepared by mixing PSS (4 mL, 2 mg/mL in DI water), an aqueous solution of Na_2CO_3 (48 μL, 1 M), and an aqueous solution of $CaCl_2$ (96 μL, 1 M) under vigorous stirring for 40 s, incubating for 7 min, and calcinating at 450 °C for 2 h. ESMH-CM shells were formed on the resulting $CaCO_3$ particles with a 1:1 mixture of the ESMH and CM stock solutions (500 μL each).

Single-Cell Nanoencapsulation (SCNE). A single colony of *S. cerevisiae*, picked from the YPD agar plate, was cultured for 30 h in a YPD broth medium at 33 °C. After washing with DI water, *S. cerevisiae* were immersed for 3 h in a 1:1 mixture of the ESMH and CM stock solutions (500 μL each) and washed with DI water three times. The same protocol was employed for *L. acidophilus* and *L. brevis*, after culturing for 24 h in an MRS broth medium at 33 °C. For viability assay of *S. cerevisiae*, 5 μL of the FDA stock solution (10 mg/mL in acetone) and 2 μL of an aqueous PI stock solution (1 mg/mL) were added to a *S. cerevisiae* suspension (1 mL), and the resultant was incubated for 15 min at 33 °C. SYTO 9 was used instead of FDA for *L. acidophilus* and *L. brevis.* To a cell suspension (1 mL) were added 2 μL of the SYTO 9 stock solution (3.34 mM in DMSO) and 2 μL of the PI stock solution (20 mM in DMSO), and the resultant was incubated for 20 min at 33 °C. To form the ESMH-CM[Fe^{3+}] shell, *L.acidophilus*@ESMH-CM or *L.brevis*@ESMH-CM was immersed in an aqueous solution of $FeCl_3$ (10 mM) for 30 min. The $t_{-2.0}^{\mathrm{OD}_{600}}$ values were calculated based on the results of cell culture in the MRS broth medium. In short, 1 mL of an aqueous cell suspension (*L. acidophilus*, *L.acidophilus*@ESMH-CM, or *L.acidophilus*@ESMH-CM[Fe^{3+}], OD_{600} = 0.15) was added to 150 mL of the MRS broth medium (final OD_{600} = 0.001) and incubated at 33 °C. The 100 μL of the culture mixture was picked at the predetermined time, and the cell density was measured at 600 nm with a microplate reader. Linear fitting of $\ln(\mathrm{OD}_{600})$, from -4.0 to 1.0, with incubation time (in hour) gave $t_{-2.0}^{\mathrm{OD}_{600}}$, the time for $\ln(\mathrm{OD}_{600})$ of -2.0.

Cytoprotection. *(a) PEI: S. cerevisiae* and *S.cerevisiae*@ESMH-CM were suspended in 1 mL of an aqueous PEI solution (0.5, 1, 10, or 50 mg/mL) for 30 min, and the cell viability was measured by the FDA-PI assay. *(b) TA: S. cerevisiae* or *S.cerevisiae*@ESMH-CM were suspended in 1 mL of an aqueous TA solution (1, 5, 10, 25, or 50 mg/mL) for 1 h, and the cell viability was measured by the FDA-PI assay. *(c) SGF:* The SGF was made to contain 0.2% (w/v) NaCl and pepsin (3 mg/mL), with pH adjustment to 2 by 1 M HCl. The cells were suspended in the SGF solution and incubated at 37 °C for 2 h, and the cell viability was measured with SYTO 9 and PI.

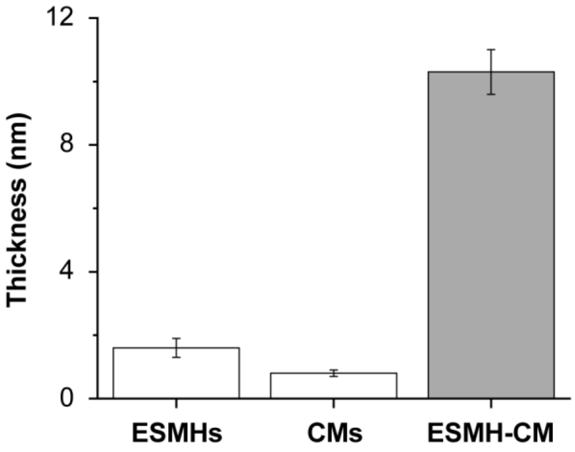

Figure S1. Graph of film thickness after 3 h of incubation (ESMHs, CMs, and ESMH-CM).

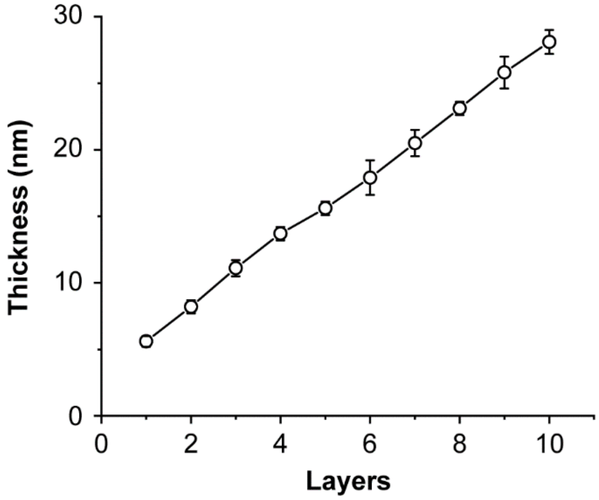

Figure S2. Thickness of [ESMH-CM]$_n$ films versus number of depositions. Each deposition was done for 10 min in a freshly made, 50-mM NaCl solution of ESMHs and CMs ([ESMH] = [CM] = 1 mg/mL).

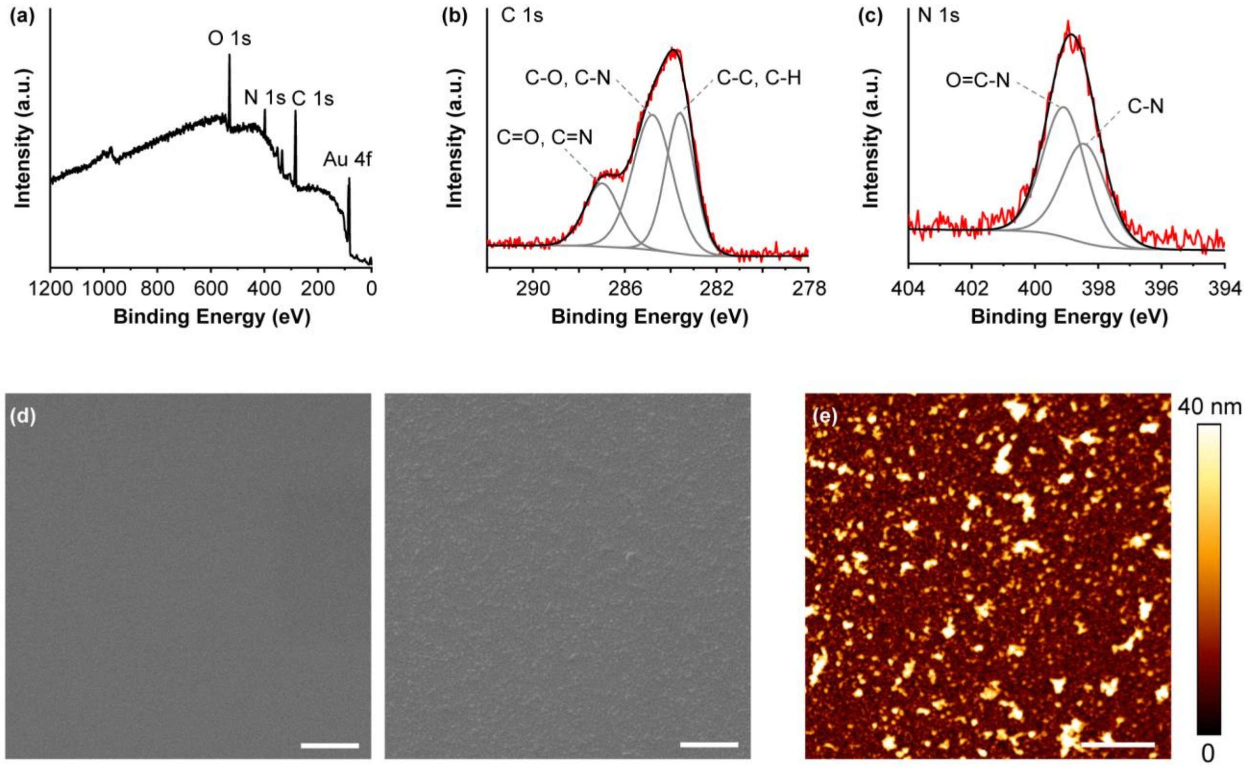

Figure S3. Characterizations of ESMH-CM films on gold substrates ([ESMH] = [CM] = 1 mg/mL; [NaCl] = 50 mM; 3 h of incubation): (a) XPS spectrum. (b-c) Deconvoluted XPS spectra for C 1s and N 1s. (d) FE-SEM images (left: bare gold substrates, right: ESMH-CM films on gold substrates). (e) AFM image. Scale bar: 1 μm.

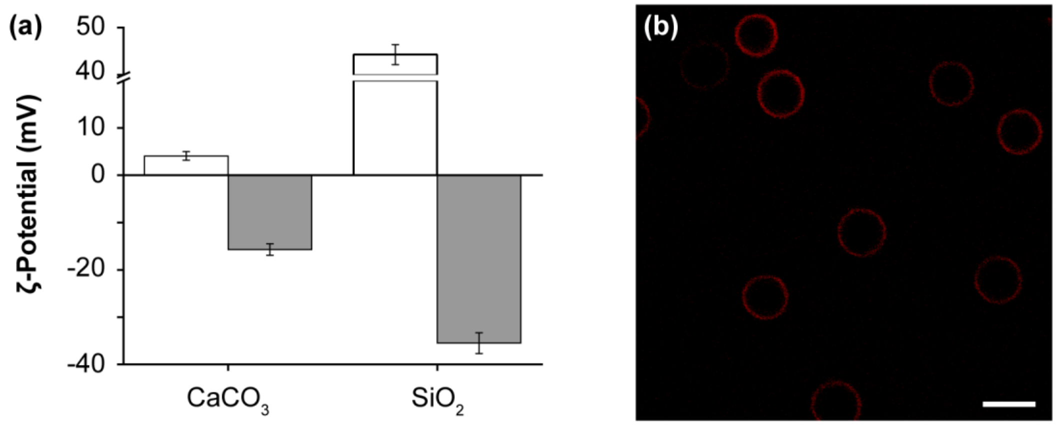

Figure S4. (a) ζ-Potential changes (white) before and (gray) after ESMH-CM-shell formation on $CaCO_3$ and SiO_2 particles. (b) CLSM image of ESMH_TAMRA-CM shells on SiO_2 particles. Scale bar: 5 μm.

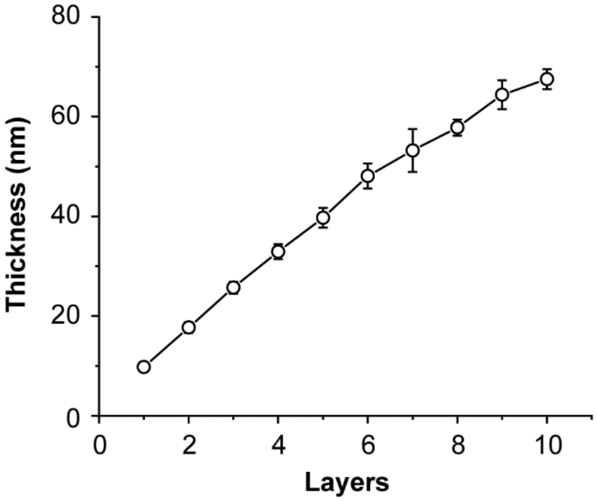

Figure S5. Thickness of [ESMH-CM]$_n$ films versus number of depositions. Each deposition was done for 3 h in a freshly made, 50-mM NaCl solution of ESMHs and CMs ([ESMH] = [CM] = 1 mg/mL).
